# A novel bispecific c-MET/CTLA-4 antibody targetting lung cancer stem cell-like cells with therapeutic potential in human non-small-cell lung cancer

**DOI:** 10.1042/BSR20171278

**Published:** 2019-05-31

**Authors:** Jian-feng Li, Yuan-yuan Niu, Yan-li Xing, Feng Liu

**Affiliations:** 1 Department of Radiation Oncology, First Affiliated Hospital, Guangzhou Medical University, Guangzhou, 510180, Guangdong Province, People’s Republic of China; 2 Department of Respiratory Medicine, The Eastern Hospital of The First Affiliated Hospital, Sun Yat-sen University, Guangzhou, 510180, Guangdong Province, People’s Republic of China; 3 Department of Geriatrics, National Key Clinical Specialty, Guangzhou First People’s Hospital, Guangzhou Medical University, Guangzhou, 510180, Guangdong Province, People’s Republic of China

**Keywords:** bispecific antibody, CTLA-4, c-MET, CD166, lung cancer stem cell-like cells, non-small cell lung cancer

## Abstract

A novel paradigm in tumor biology suggests that non-small-cell lung cancer (NSCLC) growth is driven by lung cancer stem cell (LCSC) like cells, but t here are still not any effective strategies to remove LCSCs. The bispecific antibody (BsAb) is a novel antibody, which can target two different antigens and mediate specific killing effects by selectively redirecting effector cells to the target cells. Here, we designed and synthesized a new BsAb, BsAb-5, that can target cellular mesenchymal-to-epithelial transition factor (c-MET) and cytotoxic T-lymphocyte-associated protein 4 (CTLA-4) in CD166^+^ LCSCs with high affinity and specificity, for the first time. We showed that BsAb-5 could inhibit hepatocyte growth factor (HGF) mediated tumor development, including proliferation, migration, and apoptosis, serving as an inhibitory c-MET antibody. Moreover, we demonstrated that mechanisms responsible for BsAb-5 in CD166^+^ LCSCs included inducing c-MET degradation and inhibition of HGF-stimulated c-MET-Notch pathway by using AdHGF infection, nuclei location, and Western blot assays. *In vivo*, xenograft analysis revealed that mice on BsAb-5 group showed significantly reduced tumor volume. At the meantime, the observed antitumor effects of BsAb-5 were dependent on considerably suppressing T-regulatory cells (T_regs_) and up-regulating effector T cells. On the basis of these results, we have identified a potential BsAb drug, which can effectively target c-MET and CTLA-4 in CD166^+^ LCSCs for the treatment of human NSCLC.

## Introduction

Non-small-cell lung cancer (NSCLC) remains a major cause of cancer-related deaths worldwide [1]. Despite numerous advances in our knowledge of NSCLC, development of clinical effective therapies has met with limited success [2]. It underscores the urgent need for refined investigation on a viable therapeutic option for advancing anticancer therapy.

Nowadays, accumulating evidence have shown a subpopulation of cancer cells with stem cell-like features, including self-renewal, differentiation, tumorigenesis, and tumor heterogeneity. This subpopulation of cancer cells was named cancer stem cell (CSC) like cells [3,4]. CSCs are reported as the major source of tumor recurrence after radiation or chemotherapy [5]. Recent studies have reported that CSCs exist in a variety of human malignancies [6–8]. Amongst them, the percentage of NSCLC stem cell-like cells (lung cancer stem cells (LCSCs)) following chemotherapy is increased compared with that before therapy [4]. Residual LCSCs, which chemotherapy fails to kill, will eventually lead to subsequent disease recurrence of NSCLC patients. Nevertheless, there are still not any effective strategies helping remove LCSCs.

Cellular mesenchymal-to-epithelial transition factor (c-MET), affecting cancer cell morphogenesis, angiogenesis, and cytoprotection *in vitro*, is a high-affinity receptor for binding to hepatocyte growth factor (HGF) [9]. Up-regulation of c-MET expression contributes to promoting tumor progression, invasion, metastasis, and angiogenesis in different types of solid tumors, including NSCLC [10,11]. Furthermore, activation of the HGF/c-MET pathway in various solid tumors can stimulate lymph angiogenesis, leading to lymph node metastasis [12]. At the present age, commercial c-MET inhibitors have emerged for phase II clinical trials to prolong survival of patients with hepatocellular carcinoma [13]. Nevertheless, these c-MET inhibitors are likely to carry potential side effects, such as cardiac muscle denaturation, heart rate acceleration, or body weight reduction [14,15]. Currently, monoclonal antibodies (mAbs) against growth factors or their receptors stated to be accepted as an alternative strategy for cancer therapy [16,17]. However, there are no studies using mAbs to target CSCs.

Cytotoxic T-lymphocyte-associated protein 4 (CTLA-4) is an essential negative regulator of T-cell responses [18], resulting in T-cell exhaustion and hence a state of T-cell dysfunction in many types of cancer [19]. The blockade of CTLA-4 may provide a promising adoptive cell therapy (ACT) for antitumor T-cell immunity [20]. Ipilimumab, an IgG1-blocking CTLA-4 signaling, was approved for the treatment of metastatic melanoma in 2011. It targets CTLA-4 and prevents B-cell activation antigen B7 (CD80/CD86); thus, serving as an immune checkpoint inhibitor, which promotes APC-mediated T-cell activation and thereby increase T-cell-specific immunity including antitumor immune responses [21]. However, response rates were poor and adverse events were high, which limited the use of this therapy [22]. Moreover, there are no mAbs blocking CTLA-4 in NSCLC, at the present age.

Bispecific antibodies (BsAbs) are able to block two different antigens exerting overlapping roles in pathogenesis [23], which is a promising way to enhance antitumor immunity with synergistic effects. Here, we designed and synthesized a BsAb, BsAb-5, that can target c-MET and CTLA-4 in CD166^+^ LCSCs with high affinity and specificity. We showed that BsAb-5 could inhibit HGF-mediated tumor development, serving as an inhibitory c-MET antibody. Moreover, we demonstrated that mechanisms responsible for BsAb-5 in CD166^+^ LCSCs included inducing c-MET degradation and inhibition of HGF-stimulated c-MET-Notch pathway. *In vivo*, xenograft analysis revealed that mice on BsAb-5 group showed significantly reduced tumor volume by luciferin staining in tumor images. In the meantime, the observed antitumor effects of BsAb-5 were dependent on considerably suppressing T-regulatory cell (T_regs_) and up-regulating effector T cells.

## Materials and methods

### Animal and cell culture

Female athymic BALB/c nu/nu mice, 3–4 weeks old, obtained from HFK Bioscience (China), were maintained at the Animal Core Facility at National Key Clinical Specialty, Guangzhou First People’s Hospital, Guangzhou Medical University, under specific pathogen-free (SPF) conditions. All studies on mice were conducted in accordance with the National Institutes of Health ‘Guide for the Care and Use of Laboratory Animals’ and were approved by the ethical committee of National Key Clinical Specialty, Guangzhou First People’s Hospital, Guangzhou Medical University.

### Sample collection

Ten patients with primary breast cancer, who consecutively underwent chemotherapy at National Key Clinical Specialty, Guangzhou First People’s Hospital, Guangzhou Medical University, were enrolled in the present study from January 2009 to June 2015. Informed consent for the additional core-needle biopsy and experimental use of tumor samples was obtained from all patients, following a protocol approved by the Ethics Committee of National Key Clinical Specialty, Guangzhou First People’s Hospital, Guangzhou Medical University.

### Sphere formation and propagation

Single-cell suspensions were obtained from colorectal cancer primary tissue samples. Colorectal cancer primary tissue samples were shipped to laboratory in cold RPMI-1640 medium with penicillin/streptomycin within 1 h of removal from patients. Surgical specimens were washed with cold PBS supplemented with high doses of penicillin/streptomycin three times, chopped with a sterile blade, and incubated in 1 mg/ml collagenase II (Sigma–Aldrich, U.S.A.) for 30 min at 37°C. After incubation, the suspensions were repeatedly triturated, passed through 70-µm cell strainers (BD Falcon, U.S.A.). Recovered cells were cultured at clonal density in serum-free medium containing N_2_ (100×) supplementary (Gibco-Invitrogen, U.S.A.), 0.4% BSA (Gibco-Invitrogen, U.S.A.), 100 U/ml penicillin, 1 mg/ml streptomycin in DMEM/F12 medium (Gibco-Invitrogen, U.S.A.) and supplemented with 20 mg/ml EGF, 20 mg/ml IGF, and 20 mg/ml bFGF. Flasks non-treated for tissue culture were used to reduce cell adherence and support growth as undifferentiated tumor spheres.

The medium of all the spheres was replaced with fresh growth factors twice a week until cells started to form floating aggregates. Cultures were expanded by enzymatic digestion of spheres with TrypLE Express (Gibco-Invitrogen, U.S.A.), followed by replating of both single cells in completely fresh medium. All cells were cultured at 37°C in a 5% CO_2_ humidified incubator.

### Reagents and antibodies

Human recombinant HGF was purchased from GenScript (Nanjing, China). The c-MET kinase inhibitors JNJ38877605 and rapamycin (RAPA) were obtained from Selleckchem (Houston, TX). Antibodies used include c-MET mAb, p-MET (Tyr^1234/1235^) mAb, Notch mAb, HES-1 mAb, and GAPDH mAb. Antibodies used in this study include c-MET mAb, p-MET (Tyr1234/1235) mAb (Abcam, U.S.A.), Notch mAb (Abcam, U.S.A.), HES-1 mAb (Abcam, U.S.A.), GAPDH mAb (Abcam, U.S.A.), Anti-mouse IgG (Abcam, U.S.A.), anti-CTLA4, #EPR1476 (Abcam, U.S.A.) and anti-human IgG4(Abcam, U.S.A.). All antibodies were purchased from Cell Signaling Technology, Inc.

### Construction of the *c-MET-IgG-scFv* (CTLA-4) fusion gene

To yield cDNA-encoding hinge region and CH_2_ and CH_3_ domains of human IgG1, mRNA was extracted from PBMCs of a healthy donor using a Pharmacia QuickPrep Total RNA Extraction Kit (Amersham Biosciences, Freiburg, Germany), and cDNA was synthesized using the Pharmacia First-strand cDNA Synthesis Kit (Amersham Biosciences). Primers IgG1-FOR (5′-aaacgctagcatcgatcctaggagAGCCCAAATCTTCTGACAAAACTCACACATGCCC-3′) and IgG1-BACK (5′-tttgaagcTTACCCGGAGACAGGGAG AGGC-3′; IgG1 sequence in upper case) were employed in PCR using the PBMC cDNA as template to amplify the IgG1 cDNA, and to introduce an NheI-ClaI-AvrII polylinker and a HindIII restriction site at its 5′ and 3′ ends, respectively. The amplified IgG1 cDNA fragment was digested with NheI and HindIII and inserted in frame 5′ of the ErbB2-specific scFv (CTLA-4) single-chain antibody domain, a synthetic sequence encoding the Myc-tag, recognized by mAb 9E10, and a cluster of six histidine residues (His-tag) in a modified pBluescript KS + vector (Stratagene, Heidelberg, Germany). The final *c-MET-IgG-scFv* (CTLA-4) fusion gene was assembled by inserting the c-MET domain-encoding amino acid of mature human c-MET, as an NheI/ClaI fragment at 5′ of the IgG-scFv (CTLA-4)-Myc-His sequence.

For expression in the yeast *Pichia pastoris* (*P. pastoris*), an NheI/NotI c-MET-IgG-scFv (CTLA-4) fragment including Myc- and His-tags was isolated from the cloning vector and inserted into AvrII/NotI-digested yeast expression vector pPIC9K (Invitrogen), resulting in plasmid pPIC9K-c-MET-IgG-scFv (CTLA-4). For expression of a control protein lacking the c-MET domain, plasmid pPIC9K-IgG-scFv (CTLA-4) was constructed by inserting an NheI/NotI IgG-scFv (CTLA-4)-Myc-His fragment derived from the intermediate pBluescript cloning vector into AvrII/NotI-digested pPIC9K. All PCR products and plasmid construct were verified by DNA sequencing using an ABIPrism 310 Genetic Analyzer (Applied Biosystems, Langen, Germany).

### Expression and purification of c-MET-IgG-scFv (CTLA-4)

The pPIC9K-derived expression plasmid encodes, under control of the methanol-inducible AOX1 promoter, the c-MET-IgG-scFv (CTLA-4) molecule fused to an N-terminal yeast a-factor secretion signal. In addition, the plasmid contains a functional histidinol dehydrogenase (HIS4) gene for positive selection in the *P. pastoris* HIS4 mutant strain GS115 (Invitrogen). pPIC9K-c-MET-IgG-scFv (CTLA-4) was linearized by SalI digestion and used for transformation of *P. pastoris* GS115 cells by electroporation following the manufacturer’s recommendations. His4 C yeast colonies that had restored histidinol dehydrogenase activity by stable integration of the expression plasmid into the yeast genome upon recombination were isolated using minimal dextrose medium without histidine as selection medium. Presence of the c-MET-IgG-scFv (CTLA-4) expression cassette was verified by PCR using primers AOX1 5′ (Invitrogen) and IgG1-BACK. Protein expression levels of positive clones were tested by examining the supernatants of small-scale cultures in buffered methanol complex medium. Culture supernatants were analyzed by SDS/PAGE and immunoblotting. Recombinant proteins were detected either with murine mAb 9E10 prepared from hybridoma supernatant or polyclonal rabbit anti-human IgG antibody (DAKO, Hamburg, Germany) followed by species-specific HRP-conjugated secondary antibodies (Sigma–Aldrich, Munich, Germany) and chemiluminescent detection with the ECL kit (Amersham Biosciences). Pichia clones showing the highest protein expression were used for scale-up.

For preparative expression of recombinant c-MET-IgG-scFv (CTLA-4), a single yeast colony was grown in baffled flasks, agitating at 200 rpm, to an absorbance A_600_ nm less than six in buffered glycerol complex medium (pH 6). To keep cells in the exponential growth phase at low density, repeatedly fresh medium was inoculated with small aliquots from the previous cultivation step with A_600_ nm never exceeding six. In addition, to limit protein degradation, propagation and expression were carried out at room temperature. Gene expression from the AOX1 promoter was induced by exchanging the medium with buffered methanol complex medium (pH 8, 3% methanol). Then expression cultures were kept in baffled flasks (200 rpm) for 72–90 h with replenishment of methanol (1% final concentration) after days 1 and 3, or daily. Subsequently, yeast cells were removed by centrifugation at 15000 ***g*** at 29910 rpm. Supernatants containing soluble c-MET-IgG-scFv (CTLA-4) protein were passed through a 0.22 mm filter (Millipore GmbH, Schwalbach, Germany) and applied on to a 1 ml HiTrap Protein-G column (Amersham Biosciences, Freiburg, Germany) using an AKTA FPLC (Amersham Biosciences). After binding the column was washed with 10–15 column volumes PBS (pH 7.4), before bound protein was eluted in a single step with 100 Mm glycine (pH 2.7). Eluates were neutralized by adding 10% fraction volume of 1 M Tris/HCl (pH 8.8), and subsequently dialyzed against PBS (pH 7.4). Purity and integrity of c-MET-IgG-scFv (CTLA-4) were determined by SDS/PAGE and Coomassie staining, or immunoblotting with mAb 9E10 or anti-human IgG antibody. Purified protein was stored at −20°C. IgG-scFv (CTLA-4) control protein lacking the c-MET domain was produced following similar procedures.

### Cell viability and apoptosis assay

Cells were seeded in a 96-well plate at 1 × 10^4^ each hole overnight and were grown in the presence of IgG4 (control) and BsAb for 8 h and JNJ for 2 h, then were treated with HGF. After 3 days, 20 μl MTS (Sango, China) was added to each sample and incubated for 4 h. The absorbance of solution was recorded at 490 nm with a thermo microplate reader. The results of the MTS assay to reflect cell viability. Apoptotic cells were measured by flow cytometry as follows: cells were harvested and washed with PBS, resuspended in prediluted binding buffer, and stained with annexin V-FITC (BD Biosciences, CA, U.S.A.) for 30 min at room temperature. After being washed and resuspended in PI-binding buffer, the cells were immediately subjected to apoptosis analyses by flow cytometry using Cell Quest Software.

### RNA isolation and real-time quantitative PCR

Total RNA was isolated from each cell line with TriReagent (Sigma–Aldrich) according to the manufacturer’s instructions. cDNA synthesis and quantitative PCR (qPCR) reactions were performed with SYBR^®^ FAST qPCR Kit Master Mix (KAPA). The primer pairs used were c-MET, forward 5′-TTC-ACC-GCG-GAA-ACA-CCC-ATC-3′, reverse 5′-GTC-TTC-CAG-CCA-GGC-CCA-3′; and GAPDH, forward 5′-CAT-CTC-TGC-CCC-CTC-TGC-TGA-3′, reverse 5′-GGA-TGA-CCT-TGC-CCA-CAG-CCT-3′. Cycle threshold (Δ*C*_T_) values were calculated based on the mean *C*_T_ values of the target genes and mean *C*_T_ values of the reference control gene *GAPDH*, using the following formula: Δ*C*_T_ = mean *C*_T_ for target gene – mean *C*_T_ for GAPDH. Relative gene expression levels were calculated using ΔΔ*C*_T_ analysis. ΔΔ*C*_T_ = Δ*C*_T_ of sample – Δ*C*_T_ of calibrator. Relative gene expression =2^−ΔΔ*C*_T_^.

### Western blotting

To determine the molecular mechanism of HGF on c-MET signaling pathway, Western blot analysis was performed to detect key proteins involved the pathway. The cells were lysed with M-PER^®^ Mammalian Protein Extraction (Pierce). Proteins were quantitated by the BCA Protein Assay kit (Pierce) according to the manufacturer’s instructions. Samples containing a total of 50 μg protein were incubated at 100°C for 5 min, separated by SDS/PAGE, and subsequently electrotransferred on to a PVDF membrane. Essential component detection in the cells was performed with antibody at overnight incubation at 4°C, and then HRP-conjugated secondary antibody (1:5000 dilution; Pierce Chemical) was added for 1 h at room temperature, followed by the development of reactions in a chemiluminescent detection system. GAPDH antibody was used as the control.

### Migration assays

For the migration assay, cells were plated in six-well plates at a density of 3 × 10^5^ and incubated in DMEM-0% FBS (serum-free medium) in the presence of BsAb for 8 h and JNJ for 2 h, then were treated with HGF (100 ng/ml). Cells were resuspended in serum-free medium and seeded into transwell chambers with 8-μm diameter pore size polycarbonate membranes (Corning, NY, U.S.A.). Medium containing 30% FBS was used in the lower chamber as an attractant. After 12 h, cells on the upper chamber surface were removed using cotton swabs. The migrated or invaded cells in the lower surface were fixed with 4% paraformaldehyde and stained with 0.1% Crystal Violet. The total numbers of cells were captured and analyzed from ten different fields with the Olympus IX2 microscope.

### Flow cytometry assay and FACS

Flow cytometry assay was done on single-cell suspensions obtained by enzymatic digestion of spheres which were derived from primary NSCLC samples and labeled with FITC-conjugated anti-CD166 antibody (Abcam, U.S.A.) by using an Epics Altra flow cytometer (Beckman Coulter, U.S.A.).

FASC spheres, which were derived from primary tumor samples, were dissociated into single cells by enzymatic digestion. Single-cell suspensions were washed and incubated in staining solution of 1% BSA and 2 mM EDTA with the specific antibodies at appropriate dilutions.

### Human PBMCs preparation and transplantation

Blood from healthy volunteers was collected in heparinized tubes. For the isolation of PBMCs, blood was diluted 1:1 with RPMI-1640 medium (vol/vol) prior to transferring into the leucosep tube. Following centrifugation (20 min, 2000 rpm), the PBMC layer was pooled and transferred into a 15 ml falcon tube. The sample was washed with 10 ml PBS and centrifuged again for 10 min at 1500 rpm. The obtained cell pellet was resuspended in PBS. A total of 1 × 10^8^ PBMCs per mouse were injected into NOD/SCID mice through tail vein for the reconstitution of immune system.

### Tumor xenograft study

Approximately 5- to 6-week-old male NOD/SCID mice (~20 g) were obtained from Hua Fu Kang Co., Ltd. (Beijing, China) and were kept in a SPF facility. They are absent from T, B lymphocytes, and NK cells. All animal treatments were in accordance with international ethics guidelines and the National Institutes of Health Care and Use of Laboratory Animals. The present study was approved by Guangzhou First People’s Hospital, Guangzhou Medical University. LCSCs (10^4^ cells) were subcutaneously injected into the right flank region of mice. After 7 days, allowing the tumors to grow ~50 mm^3^, mice were randomized into control and treatment groups. For LCSCs xenograft, mice were treated as follows: BsAb (10 mg/kg, twice per week), anti-CTLA4 (#EPR1476, Abcam) (10 mg/kg, twice per week); PBMCs (1 × 10^8^) were injected the first and fourth time drugs were administered via tail vein. Each group consisted of three to four mice. At the same time, body weight and tumor size were measured using an electronic balance and a vernier caliper. Tumor volume was calculated using the formula: volume = (length × width^2^) × 0.5. After the endpoint (28 days after the implantation), blood was collected from the aorta abdominalis and the serum was separated and frozen at −80°C for further analyses. Mice were then killed for the collection of tumors.

### Bioluminescent imaging of tumors in live mice

Luciferase-expressing tumors were detected by IVIS-200 Imaging System (Xenogen Corporation, Alameda, CA, U.S.A.). Briefly, mice were injected by intraperitoneally (IP) with D-luciferin (150 mg/kg, Thermo Fisher Scientific, Waltham, MA, U.S.A.) after being anesthetized. Images were acquired with the IVIS-200 Imaging System at appropriate exposure times, the imaging and quantitation of signals were analyzed by the Living Image software with proper background subtraction.

### Statistical analysis

All data are expressed as mean values ± S.D., except cell cycle data, which are presented as means. All statistics were analyzed with SPSS 20.0 software package (SPSS Inc., Chicago, IL, U.S.A.). One-way ANOVA, followed by Bonferroni post-hoc comparison tests (in case of equal variances) or Welch and Brown–Forsythe tests (in case of unequal variances), was performed for comparisons amongst control and treatment groups. *P*<0.01, ** was considered statistically significant.

## Results

### CD166^+^ spheres from primary NSCLC samples and cell lines display stem cell-like features

**Figure 1:**
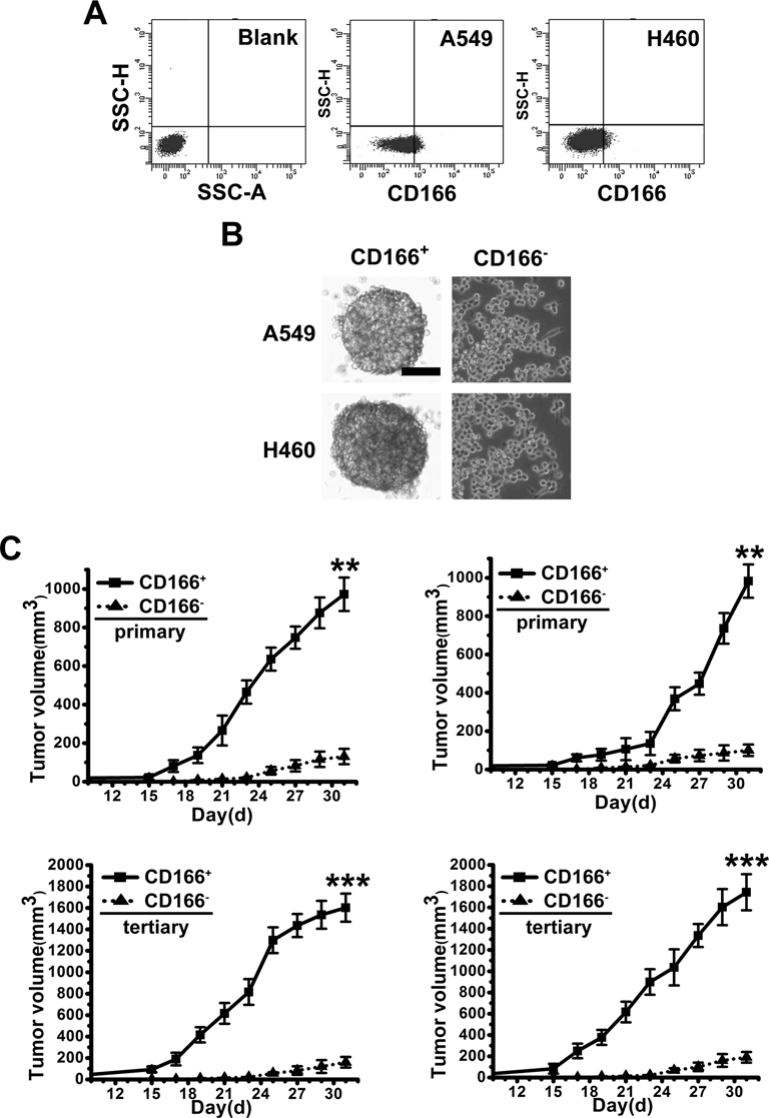
CD166^+^ spheres from NSCLC primary tissue samples and cell lines display stem cell-like features (**A**) Flow cytometer analysis of CD166 expression in fresh NSCLC primary tissue samples and cell lines (A549 and H460). (**B**) Phase-contrast images of CD166^+^ sphere cells and CD166^−^ cells. Scale bar: 50 μm. (**C**) *In vivo* serial transplantation assay. A total of 10^4^ CD166^+^ cells and CD166^−^ cells, purified from A549 and H460, were injected s.c. into nude mice. Derived tumor xenografts were dissociated to single-cell suspension and then serially re-injected in mice (10^4^ cells), generating secondary and then tertiary tumors. Tumor growth curves of primary and tertiary tumors are shown. Columns, mean of three individual experiments; S.D., ***P<0.001; S.D.,**P<0.01.

CSCs are believed to be able to form spheres in serum-free cultivation. We cultured primary NSCLC tissue cells and two NSCLC cell line cells, A549 and H460, to induce sphere formation in serum-free cultivation. After culturing for 2–4 weeks, even though a large number of cells died, some tumor cells grew to form spheres (results not shown). To identify whether CD166 is the surface marker to sort LCSCs, we analyzed ten fresh tumor samples derived from a series of NSCLC patients. Flow cytometry demonstrated the presence of a variable fraction of CD166^+^ cells, ranging from 0.1 to 1.2%, in nine out of ten NSCLC specimens (Figure 1A; Supplementary Table S1). Then, we fractionated CD166^+^ and CD166^−^ cells by FASC for further study (Figure 1A). All of CD166^+^ cells could form spheres in serum-free cultivation, CD166^+^ spheres were at least passaged ten times, indicating the self-renewal of these sphere cells (Figure 1B). However, just a few CD166^−^ cells could form compact spheres and could not be passaged in serum-free cultivation (Figure 1B). Additionally, we also demonstrated that almost all of CD166^+^ cells expressed CD133 (Supplementary Figure S1) which is generally believed as the maker of CSCs. To evaluate the tumorigenic potential of CD166^+^ spheres, the same numbers of CD166^+^ and CD166^−^ cells (10^4^ cells) were subcutaneously injected in the flank of nude mice. Tumor formation of CD166^+^ spheres was faster and resulted in increased tumor take compared with that observed in CD166^−^ cells (Figure 1C). To investigate whether *in vivo* tumors originating from CD166^+^ spheres possess long-term tumorigenic potential, we performed serial transplantation assays in nude mice of cells isolated from tumor xenografts originally derived from CD166^+^ or CD166^−^ cells injection. Cells derived from CD166^+^ tumors were able to generate tumors in primary, secondary, and tertiary transplantation. Whereas cells from CD166^−^ tumors lost tumorigenic potential during serial transplantations (Figure 1C).

### Both of c-MET and CTLA-4 are overexpressed in CD166^+^ LCSCs from primary NSCLC tumor tissues and cell lines

**Figure 2:**
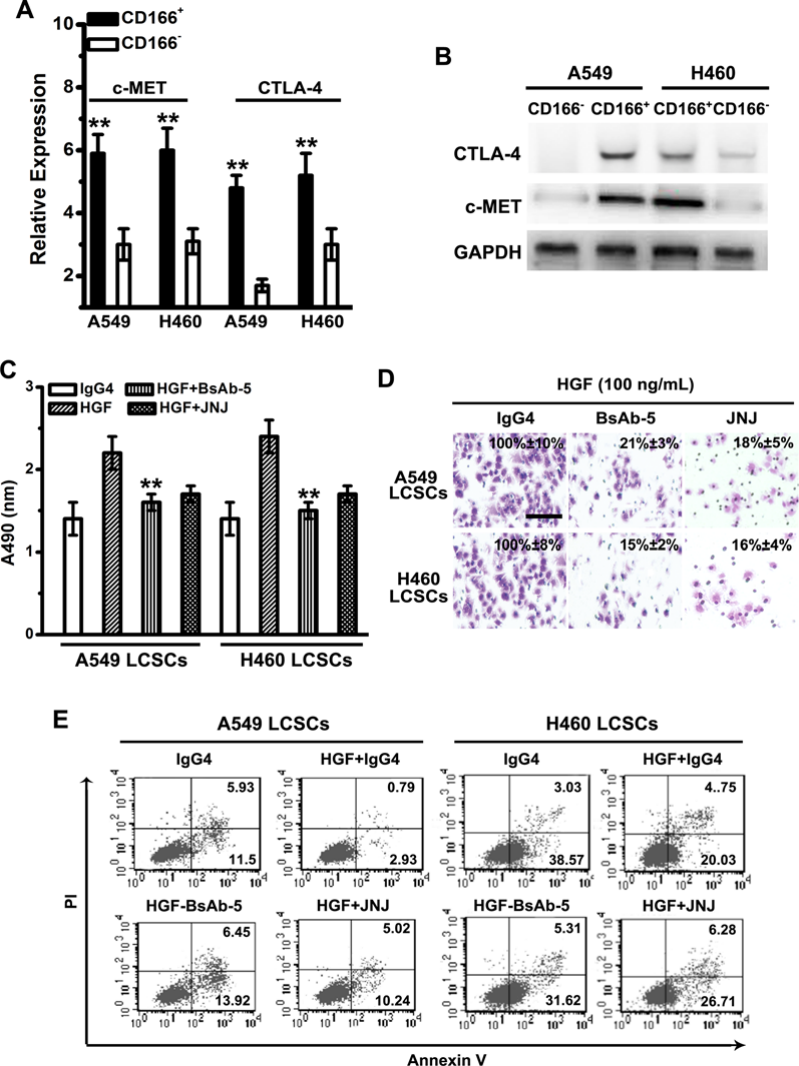
BsAb-5 inhibits HGF-stimulated CSC-like phenotype and tumor development *in vitro* (**A**) c-MET and CTLA-4 mRNA expressions were detected by qPCR. Note: columns, mean of three individual experiments; S.D.,**P<0.01. (**B**) c-MET and CTLA-4 protein expressions were detected by Western blot analysis. GAPDH expression was used as an internal control. (**C**) The viability of CD166^+^ LCSC cells was assessed by MTS assay after treatment with HGF (100 ng/ml), IgG4 (0.5 μM), BsAb (0.5 μM), and JNJ (0.5 μM) for 72 h. Columns, mean of three individual experiments; S.D., **P<0.01. (**D**) Transwell assays without matrigel were used to detect the migration activity in different group. Each experiment was repeated three times. Scale bar: 100 mm. (**E**) CD166^+^ LCSCs were incubated with BsAb (0.5 μM) for 8 h or JNJ (0.5 μM) for 2 h and then treated with combinations of HGF (100 ng/ml) and RAPA. After 48 h treatment, apoptotic cells stained with annexin V and propidium iodide, and analyzed by flow cytometry. Columns, mean of three individual experiments; S.D., **P<0.01.

qPCR assay was used to evaluate the expression of c-MET and CTLA-4 in primary NSCLC tumor tissues. Both c-MET and CTLA-4 are up-regulated in NSCLC compared with those in adjacent normal tissues (Supplementary Table S1). Meanwhile, CD166^+^ LCSCs from A549 and H460 expressed a relatively higher levels of c-MET and CTLA-4 than their CD166^−^ counterparts (Figure 2A,B).

### BsAb-5 inhibits HGF-stimulated tumor development in LCSCs via c-MET-Notch pathway

Through a series of assays, including EC50 testing, competitive ELISA testing, SPR, and epitope mapping, we found BsAb-5 which can target c-MET and CTLA-4 in CD166+ LCSCs with high affinity and specificity (Supplementary Table S2). To evaluate whether BsAb-5 is able to block c-MET to affect the proliferation, migration, and apoptosis, we used JNJ38877605 (for 2 h), a known c-MET inhibitor. At the meantime, HGF, and BsAb-5 (for 8 h) were also used to pretreat A549 and H460 LCSCs. The cell proliferation was significantly decreased in HGF + BsAb-5 group, as well as that in HGF + JNJ group (Figure 2C). Likewise, BsAb-5, as well as JNJ, considerably inhibited the migration of A549 and H460 LCSCs (Figure 2D). Furthermore, A549 and H460 LCSCs treated with BsAb for 8 h or JNJ for 2 h, and then treated with combinations of HGF and RAPA for 48 h were used to test the effect of BsAb-5 on c-MET-mediated signaling in apoptosis. In the presence of BsAb-5 and JNJ, LCSCs showed increased percentage of Annexin V^+^PI^+^/Annexin V^+^PI^−^ cells (Figure 2E). Thus, these results indicate that BsAb-5 inhibits HGF-mediated proliferation, migration, and apoptosis, serving as an inhibitory c-MET antibody.

**Figure 3:**
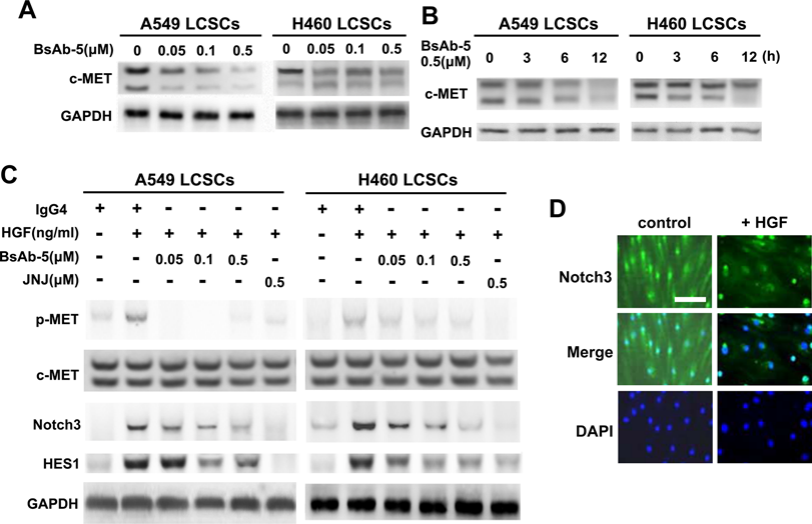
BsAb-5 in CD166^+^ LCSCs induces c-MET degradation and inhibition HGF-stimulated c-MET-Notch pathway (**A,B**) Cells were treated with the indicated concentrations of BsAb-5 for 24 h (A) or for the indicated time (B). (**C**) CD166^+^ LCSCs were treated with the indicated concentrations of BsAb-5 for 8 h or JNJ for 2 h, and then stimulated with or without HGF (100 ng/ml) for 30 min. Total cell lysates were evaluated by Western blot using specific antibodies. GAPDH expression was used as an internal control. (**D**) Representative micrographs show localization of active Notch3 in CD166^+^ LCSCs infected with control AdEGFP or AdHGF. Scale bar: 10 μm.

By exploring whether BsAb-5 inhibits HGF-triggered c-MET molecules, BsAb-5 was found to down-regulate c-MET protein expression in both dose- and time-dependent manners in LCSCs by Western blot assay (Figure 3A,B), which suggests that BsAb-5 could induce c-MET degradation. Furthermore, up-regulation of Notch3 and HES1, in response to HGF stimulation, were inhibited by BsAb-5 and JNJ (Figure 3C). Thus, these results suggest that BsAb-5 inhibits HGF-stimulated c-MET-Notch pathway and induces c-MET degradation. Moreover, we interrogated Notch3 signaling during HGF-induced LCSCs. Eight days after AdHGF infection of LCSCs. However, Notch3 was detected in both the cytoplasm and nuclei after AdHGF infected LCSCs incubated with BsAb-5, Notch3 was mainly localized in the nuclei, suggesting HGF overexpression triggers Notch3 activation (Figure 3D).

### BsAb-5 contributes to antitumor effects by suppressing T_regs_ and up-regulating effector T cells

**Figure 4:**
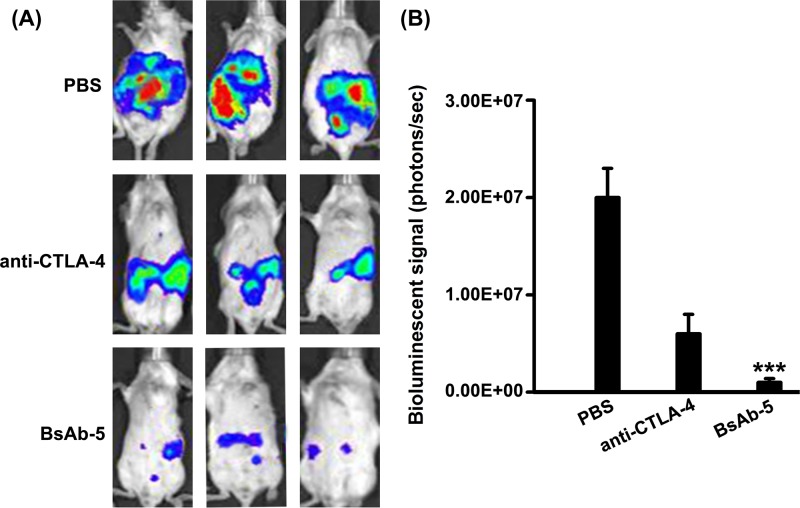
BsAb-5 contributes to antitumor effects *in vivo* (**A**) Representative bioluminescence images of mice from each group are shown (day 10 post-treatment). Stable luciferase-Renca cells were detected using the IVIS-200 Imaging System. (**B**) Each tumor volume was detected via the IVIS-200 Imaging System. Data were analyzed amongst groups (*n*=3). Columns, mean of three individual experiments; S.D., ***P<0.001.

To appraise the effect of BsAb-5-blocking CTLA-4, we applied it in murine model *in vivo*. Mice were randomly divided into four groups and received treatment as outlined in the ‘Materials and methods’ section. Tumor volumes were detected by the IVIS-200 Imaging System on day 10 post treatment (Figure 4A). The photo flux indices were analyzed amongst groups. When compared with mice treated with PBS, mice on anti-CTLA-4 and BsAb-5 showed significantly reduced tumor volume (Figure 4B).

**Figure 5:**
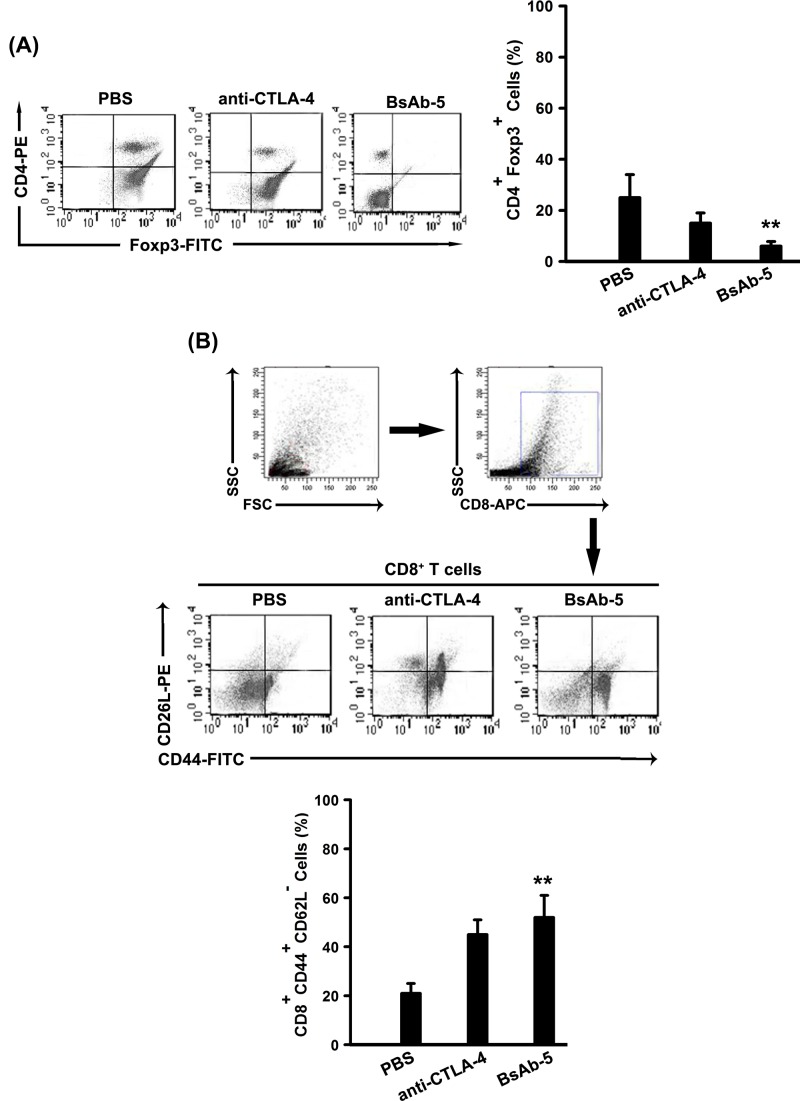
BsAb-5 suppresses T_regs_ and up-regulates effector T cells (**A**) Blood samples from mice (control and treatment groups) were stained with CD4 and Foxp3 and analyzed by flow cytometry. Representative data are graphically shown. Columns, mean of three individual experiments; S.D., **P<0.01. (**B**) Blood samples from mice (control and treatment groups) were stained with CD8, CD62L, and CD44 and analyzed by flow cytometry. Representative data are graphically shown. Columns, mean of three individual experiments; S.D., **P<0.01.

T_regs_ are known to suppress immune responses toward tumors. There was a significant decrease in CD4^+^ Foxp3^+^ T_regs_ in BsAb-5 group and anti-CTLA-4 group comparing with that in control group (Figure 5A). Additionally, we also assessed the frequency of CD8^+^ CD44^+^ CD62L^−^ effector T cells by flow cytometer. We found that the number of CD8^+^ CD44^+^ CD62L^−^ effector T cells increased considerably in BsAb-5 group and anti-CTLA-4 group (Figure 5B).

## Discussion

BsAb can redirect specific immune tumor cells to enhance tumor killing and enable potentially increase binding specificity by interacting with two different cellular surface antigens instead of one; therefore, it offers a promising way of enhancing antitumor immunity in immunotherapy with the goal of achieving synergistic effects [23]. Currently, more than 30 BsAbs have so far been exploited in a clinical setting, and 2 BsAbs have been approved for the market to treat pro-B cell acute lymphocyte leukemia by the FDA [24].

Here, *in vitro*, BsAb-5 inhibits HGF-mediated tumor development, including proliferation, migration, and apoptosis, serving as an inhibitory c-MET antibody. BsAb-5 was found to down-regulate c-MET protein expression in both dose- and time-dependent manners, which suggested that BsAb-5 could induce c-MET degradation. It is well acknowledged that HGF is a mesenchyme-derived cytokine with multiple biological effects on a wide variety of cells including mitogenic, motogenic, morphogenic, and anti-apoptotic activities [25]. In particular, the HGF receptor is encoded by the Met proto-oncogene. HGF/c-Met signaling operates through many downstream targets, including PI3K/AKT and Notch in myocardium [26]. The Notch family comprises four transmembrane receptor proteins (Notch 1–4). Cleavage of the receptor leads to release and nuclear translocation of the intracellular domain (NICD), activating downstream target genes, which play important roles in NSCLC development. Furthermore, c-MET was showed to regulate Notch3-HES-1 pathway in previous studies [25]. In this study, we demonstrated that mechanisms responsible for BsAb-5 in CD166^+^ LCSCs included inducing c-MET degradation and inhibition HGF-stimulated c-MET-Notch pathway.

At the present age, immune checkpoint inhibitors CTLA-4 blockage, PD-1 and PD-L1 blockage, are currently undergoing clinical trials and show promising results [20]. Two phase III studies are investigating PD-1, CTLA-4, and PD-L1 antibodies in combination with anti-VEGF therapy for cancer patients. Nevertheless, the effectiveness of immune checkpoint inhibitors in patients with NSCLC is unknown. Therefore, developing a new therapeutic strategy with improved effectiveness is of paramount importance. Here, when compared with mice treated with PBS, mice on anti-CTLA-4 + PBMC treatment and BsAb-5 + PBMC treatment showed significantly reduced tumor volume. Meanwhile, the significant decrease in CD4^+^ Foxp3^+^ T_regs_ and the enhanced CD8^+^ CD44^+^ CD62L^−^ effector T cells in BsAb-5 group partially explain the robust antitumor effects of BsAb-5. On the other hand, some concerns raised like that, whether the NOD/SCID mice have intrinsic agonistic activity or humanized CTLA4 highly expressed in mice need further demonstration. Meanwhile, our study did not directly address the effects of BsAb comparing with one-armed c-MET antibody *in vivo*. Only through comparing with the antibody, could we conclude that the BsAb-5 is indeed superior to the one-armed CTLA4 antibody. Further studies are necessary to explore the hypothesized superiority of BsAb-5 over CTLA4 antibody *in vitro* and *in vivo*.

In conclusion, we found that BsAb-5 can target c-MET and CTLA-4 in CD166+ LCSCs with high affinity and specificity. We showed that BsAb-5 could inhibit HGF-mediated CSC-like phenotypes and tumor development, serving as an inhibitory c-MET antibody. Moreover, we demonstrated that mechanisms responsible for BsAb-5 in CD166^+^ LCSCs included inducing c-MET degradation and inhibition HGF-stimulated c-MET-Notch pathway. *In vivo*, xenograft analysis revealed that mice on BsAb-5 group showed significantly reduced tumor volume. At the meantime, the observed antitumor effects of BsAb-5 were dependent on considerably suppressing T_regs_ and up-regulating effector T cells. On the basis of these results, we have identified a potential BsAb drug, which can effectively target c-MET and CTLA-4 in CD166^+^ LCSCs for the treatment of human NSCLC.

## Supporting information

**Figure S1:** Expression of CD133 in CD166^+ ^spheres by immunofluorescence staining. Scale bar, 100 mm.

**Table S1:** Expression of CD166, CTLA-4 and c-MET in NSCLC samples

**Table S1:** The affinity and specifity of BsAb-5

## Abbreviations

BsAb: bispecific antibody
CSC: cancer stem cell
CTLA-4: cytotoxic T-lymphocyte-associated protein 4
c-MET: cellular mesenchymal-to-epithelial transition factor
HGF: hepatocyte growth factor
LCSC: lung cancer stem cell
mAb: monoclonal antibody
NSCLC: non-small-cell lung cancer
qPCR: quantitative PCR
RAPA: rapamycin
T_reg_: T-regulatory cell
