# Supplementary material for: A novel bispecific c-MET/CTLA-4 antibody targetting lung cancer stem cell-like cells with therapeutic potential in human non-small-cell lung cancer
Source: Biosci Rep. 2019 May 31;39(5):BSR20171278. doi: 10.1042/BSR20171278 (PMC6542762; doi:10.1042/BSR20171278)
Supplement: Supplementary file 1 [file bsr20171278_Supp1.pdf]

Supplemental Information

A novel bispecific c-MET/CTLA-4 antibody targeting lung cancer stem cell-like cells with therapeutic potential in human non-small cell lung cancer

Jian-feng Li [1+], Yuan-yuan Niu [2+], Yan-li Xing [2], Feng Liu [3]*

[1] Department of Radiation Oncology, The First Affiliated Hospital, Guangzhou Medical University, Guangzhou, 510180, Guangdong Province, People's Republic of China
[2] Department of Respiratory Medicine, The Eastern Hospital of The First Affiliated Hospital, Sun Yat-sen University, Guangzhou, 510180, Guangdong Province, People's Republic of China
[3] Department of Geriatrics, National Key Clinical Specialty, Guangzhou First People's Hospital, Guangzhou Medical University, Guangzhou, 510180, Guangdong Province, People's Republic of China

*** Correspondence**: Feng Liu, Department of Geriatrics, National Key Clinical Specialty, Guangzhou First People's Hospital, Guangzhou Medical University, No. 1, Fupan Road, Guangzhou, 510180, Guangdong Province, People's Republic of China.
Telephone: 020-81048559; Fax: 020-81048559; email:

[+] Jian-feng Li and Yuan-yuan Niu contributed equally to this work.

Table S1. Expression of CD166, CTLA-4 and c-MET in NSCLC samples

Case	Age	TNM	Clinical stage	Percentage of $CD166^+$ cells	[a]Normalized CTLA-4 amount $2^{-\Delta\Delta Ct}$	[a]Normalized c-MET amount $2^{-\Delta\Delta Ct}$	Sphere formation
1	42	T1N0M0	IA	0.1%	3.3600	2.3657	No
2	35	T2N0M0	IB	0.3%	4.3423	5.2657	Yes
3	54	T1N1M0	IIA	0.6%	3.2454	3.2147	Yes
4	36	T2N1M0	IIA	0.7%	5.5938	4.1139	Yes
5	57	T2N2M0	IIB	1.1%	3.3579	2.4876	Yes
6	45	T3N0M0	IIIA	1.2%	4.2974	3.2014	Yes
7	48	T3N3M1	IV	0.8%	2.4732	3.1584	Yes
8	37	T2N2M0	IIIA	0.3%	3.4923	4.6957	Yes
9	46	T2N0M0	IB	0.5%	1.9832	6.3952	Yes
10	61	T2N3M0	IIIB	0.9%	5.0394	5.3461	Yes

[a] Relative quantification was performed by the $2^{-\Delta\Delta Ct}$ method with the adjacent lung tissue sample as a calibrator. Data show the means from three independent analyses. Every independent analysis

was carried out after the RNA extraction step. Total RNA was poly-A tailed, reverse transcript, and then real-time PCR tested. ΔC_T obtained from real-time PCR was subject to paired t-test ($\Delta C_T = C_{T\ CTLA\text{-}4/c\text{-}MET} - C_{T\beta\text{-}actin}$). The expression levels of CTLA-4/c-MET in tumor tissues were significantly higher than normal tissues ($P < 0.01$)

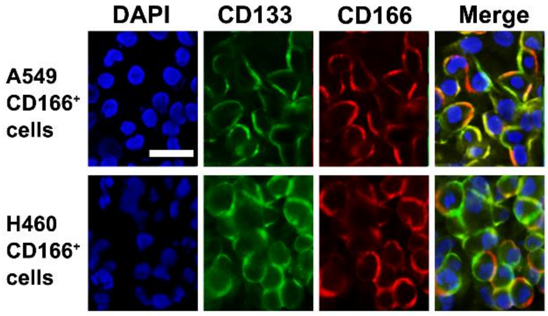

Figure S1, Expression of CD133 in CD166 $^{+}$ spheres by immunofluorescence staining. Scale bar, 100 mm.

Table S2. The affinity and specifity of BsAb-5

ID	Epitope group	Competitive Fab ELISA			EC50 (nM)	SPR (KD)
		0 nM	50 nM	5 nM		
**BsAb-5**	**A**	**100.00%**	**17.83%**	**63.21%**	**17.91**	**1.79E-09**
